# Review and Evaluate the Bioinformatics Analysis Strategies of ATAC-seq and CUT&Tag Data

**DOI:** 10.1093/gpbjnl/qzae054

**Published:** 2024-09-10

**Authors:** Siyuan Cheng, Benpeng Miao, Tiandao Li, Guoyan Zhao, Bo Zhang

**Affiliations:** Department of Developmental Biology, Center of Regenerative Medicine, Washington University School of Medicine, St. Louis, MO 63108, USA; Department of Developmental Biology, Center of Regenerative Medicine, Washington University School of Medicine, St. Louis, MO 63108, USA; Department of Genetics, Washington University School of Medicine, St. Louis, MO 63108, USA; Department of Developmental Biology, Center of Regenerative Medicine, Washington University School of Medicine, St. Louis, MO 63108, USA; Department of Genetics, Washington University School of Medicine, St. Louis, MO 63108, USA; Department of Neurology, Washington University School of Medicine, St. Louis, MO 63108, USA; Department of Pathology and Immunology, Washington University School of Medicine, St. Louis, MO 63108, USA; Department of Developmental Biology, Center of Regenerative Medicine, Washington University School of Medicine, St. Louis, MO 63108, USA

**Keywords:** Tn5 transposase, Epigenetic, Bioinformatics, ATAC-seq, CUT&Tag

## Abstract

Efficient and reliable profiling methods are essential to study epigenetics. Tn5, one of the first identified prokaryotic transposases with high DNA-binding and tagmentation efficiency, is widely adopted in different genomic and epigenomic protocols for high-throughputly exploring the genome and epigenome. Based on Tn5, the Assay for Transposase-Accessible Chromatin using sequencing (ATAC-seq) and the Cleavage Under Targets and Tagmentation (CUT&Tag) were developed to measure chromatin accessibility and detect DNA–protein interactions. These methodologies can be applied to large amounts of biological samples with low-input levels, such as rare tissues, embryos, and sorted single cells. However, fast and proper processing of these epigenomic data has become a bottleneck because massive data production continues to increase quickly. Furthermore, inappropriate data analysis can generate biased or misleading conclusions. Therefore, it is essential to evaluate the performance of Tn5-based ATAC-seq and CUT&Tag data processing bioinformatics tools, many of which were developed mostly for analyzing chromatin immunoprecipitation followed by sequencing (ChIP-seq) data. Here, we conducted a comprehensive benchmarking analysis to evaluate the performance of eight popular software for processing ATAC-seq and CUT&Tag data. We compared the sensitivity, specificity, and peak width distribution for both narrow-type and broad-type peak calling. We also tested the influence of the availability of control IgG input in CUT&Tag data analysis. Finally, we evaluated the differential analysis strategies commonly used for analyzing the CUT&Tag data. Our study provided comprehensive guidance for selecting bioinformatics tools and recommended analysis strategies, which were implemented into Docker/Singularity images for streamlined data analysis.

## Introduction

Transcriptional regulation is a critical biological process that controls when, where, and how much a gene is expressed. The dynamic interactions of proteins, such as transcription factors (TFs), cofactors, and RNA polymerase, with *cis*-regulatory DNA elements, including enhancers, silencers, and promoter elements, determine precise spatiotemporal gene expression and response to environmental stimuli. Epigenetic changes such as nucleosome position, chromatin remodeling, DNA methylation, and histone modification play critical regulatory roles in gene transcription via the modulation of protein–DNA interactions [1–8]. Alterations to these epigenetic gene regulatory mechanisms have been linked to numerous diseases, including cancer [9] and neurodevelopmental and neurodegenerative disorders [10–12]. In recent years, deciphering the epigenetic landscape has been a major focus in gaining a better understanding of the regulatory mechanisms underlying gene transcription in development and disease pathogenesis. Here, we first reviewed the major technical advances in sequencing-based high-throughput epigenetic profiling approaches for measuring chromatin status, histone modification, and TF–DNA interactions. We then provided a performance comparison of bioinformatics tools designed for analyzing sequencing data obtained by epigenetic profiling. Finally, we provided guidance for selecting tools for data processing and differential analysis, and compiled Docker/Singularity images of the recommended tools that enable streamlined data analysis by executing one line of command to yield final results.

**Figure 1:**
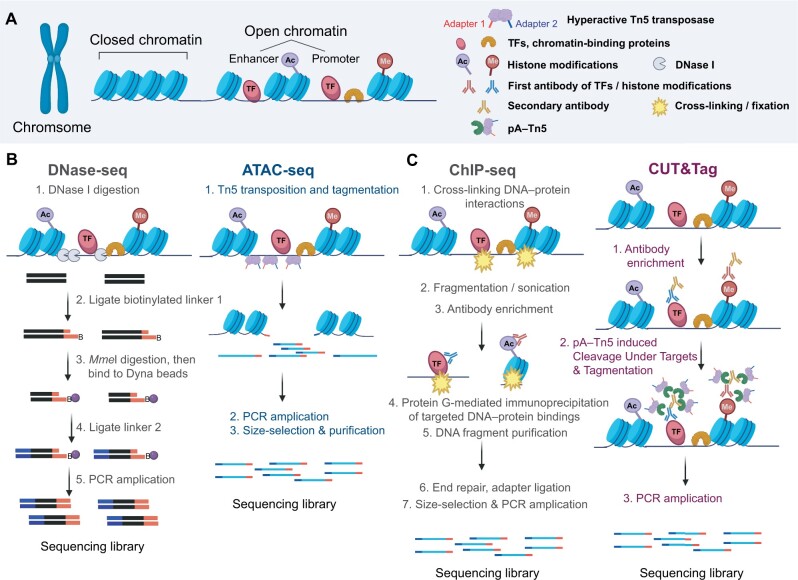
The experimental procedures of DNase-seq, ATAC-seq, ChIP-seq, and CUT&Tag **A**. Conceptualized image of chromatin accessibility. **B**. The experimental procedures of DNase-seq and ATAC-seq are performed to detect open chromatin regions. **C**. The experimental procedures of ChIP-seq and CUT&Tag are performed to detect histone modifications and TF-binding events in the genome. DNase-seq, DNase I-hypersensitive site sequencing; ATAC-seq, the assay for transposase-accessible chromatin using sequencing; ChIP-seq, chromatin immunoprecipitation followed by sequencing; CUT&Tag, the Cleavage Under Targets and Tagmentation; TF, transcription factor; PCR, polymerase chain reaction; pA–Tn5, protein A–Tn5 transposase fusion protein.

In eukaryotes, nuclear DNA is tightly packed into an array of nucleosomes. Each nucleosome consists of 147 bp of DNA wrapped in a 1.65 turn-around of a histone octamer core and is separated by linker DNA. In addition to packing genomic DNA for coiling and contraction, nucleosomes are general gene repressors of transcription. Tightly packed nucleosomes at gene promoters result in a closed chromatin state, preventing the initiation of transcription (**Figure 1**A). Open chromatin regions can be accessed by TFs and are considered regulatory elements involved in gene transcription. Specific chromatin remodeling complexes, which can slide, disassemble, or otherwise modify the configuration of nucleosomes, initiate specific positive regulatory processes. These alterations transiently expose genomic DNA, enabling it to interact with the transcriptional apparatus and leading to the expression of genes [13–15] (Figure 1A).

Eukaryotes employ a variety of mechanisms to regulate chromatin accessibility, involving dynamic interplay among histones, TFs, and chromatin remodelers. The histone proteins in the nucleosome core can be post-translationally modified by at least 80 known covalent modifications [4,5], such as acetylation, methylation, phosphorylation, and ubiquitination. Histone acetylation, a well-documented modification of chromatin, is associated with unfolding local chromatin architecture and initiating transcription. For instance, acetylation at H3K27 indicates active promoters and enhancers [1]. On the other hand, methylation is a common modification of many lysine residues in histone proteins and yields, for example, H3K4me1, H3K4me3, H3K9me3, H3K27me3, and H3K36me3. Notably, the forms and functions of histone methylation are much more diverse. For instance, H3K9me3 is a hallmark of dense, permanently condensed heterochromatin [3]. H3K4me1 and H3K4me3 are associated with active enhancers and promoters, respectively, and are marks of open chromatin regions [16]. H3K36me3 is a histone modification associated with the serine-2-phosphorylated elongating form of RNA polymerase II and is abundant mainly in the gene body [17,18].

TFs regulate chromatin accessibility through different mechanisms. Pioneer factors constitute a group of TFs that recognize target DNA sequences within quiescent chromatin and trigger remodeling of the adjoining chromatin landscape, leading to chromatin opening for increased accessibility [19]. Pioneer factors contribute to cell fate determination by inducing stable chromatin configuration alterations linked with DNA accessibility and the maintenance of epigenetic marks [19]. Most other TFs bind more efficiently to naked or nucleosome-depleted DNA [19,20]. Multiple chromatin remodeling mechanisms have been proposed to explain TF action; these mechanisms include passive competition with dynamic nucleosomes, binding to transiently accessible linker DNA, recruitment of chromatin remodelers, and stabilization of secondary TFs [20].

Regulation of chromatin accessibility is performed through chromatin remodelers. Chromatin remodelers refer to a group of ATP-dependent enzymes that regulate DNA accessibility by repositioning, ejecting, or modifying nucleosomes. Eukaryotic cells have their chromatin remodelers divided into four groups according to the ATPase subunits’ characteristics: the switch/sucrose nonfermentable (SWI/SNF) family, the imitation switch (ISWI) family, the chromodomain helicase DNA-binding (CHD) family, and the inositol requiring 80 (INO80) family [21]. Chromatin remodelers are highly conserved from yeasts to humans and play central roles in gene regulation by controlling the packing and unpacking of chromatin to regulate DNA accessibility [21].

Because of quickly evolving high-throughput sequencing technologies in recent decades, various genomic assays have been developed to characterize the epigenetic landscape and interactions between TFs and genomic DNA. To determine chromatin accessibility, DNase I-hypersensitive site sequencing (DNase-seq) was first developed in 2006 and has been widely used to identify open chromatin regions in various cell types and species [22–25]. DNase I exhibits endonuclease activity and preferentially cleaves naked DNA in open chromatin regions. A series of complicated steps are required to convert DNase I-digested fragments into sequencing-ready libraries, including the repair of DNase I-digested ends, ligation to the blunt-end of linker 1, gel purification, *Mme*I digestion, phenol and chloroform DNA extraction, ligation to phosphorylated linker 2, polymerase chain reaction (PCR) amplification of the DNase-seq products, and gel purification of precisely size-selected DNA bands [22] (Figure 1B). By sequencing a single end of a library, the exact DNase I digestion sites can be identified across the genome. These open chromatin regions enriched with DNase I digestion signals are known as DNase I-hypersensitive sites (DHSs) and associated with activated regulatory elements [26,27], such as promoters, enhancers, silencers, insulators, and locus control regions [28,29]. Due to the high sensitivity of DNase-seq, it has been adopted for studying many tissues and cell types by some genomics consortia, including the Encyclopedia of DNA Elements (ENCODE) and Roadmap Epigenomics consortia. Although DNase-seq can measure chromatin accessibility in a high-throughput fashion, an experienced scientist is needed to ensure successful outcomes.

In 2013, Buenrostro et al. [30] developed a novel assay for identifying transposase-accessible chromatins using the assay for transposase-accessible chromatin using sequencing (ATAC-seq) to measure chromatin accessibility. This experimental approach takes advantage of the highly efficient tagmentation capacity of hyperactive Tn5 transposase and involves a simple two-step experimental procedure with a low input of 500–50,000 cells (Figure 1B). Tn5 transposase preferentially inserts into open chromatin regions, whereas steric hindrance of less accessible chromatins reduces the likelihood of transposase insertion. As a result, open chromatin regions preferentially yield DNA fragments that are amenable to amplification and suitable for high-throughput sequencing techniques. ATAC-seq can be used to measure chromatin accessibility and TF footprinting simultaneously and yields a multidimensional epigenetic portrait of the gene regulation [30]. The experimental simplicity, high sensitivity and specificity, and the need for relatively few input cells quickly led to widespread ATAC-seq application in many studies. ATAC-seq has become a standard epigenetic assay to measure chromatin accessibility and map regulatory elements by several genomics consortia, such as the ENCODE, The Cancer Genome Atlas (TCGA), four-dimensional Nucleome (4DN), and Toxicant Exposures and Responses by Genomic and Epigenomic Regulators of Transcription (TaRGET) II consortia. Recently, a modified ATAC-seq method was developed to measure chromatin accessibility at the single-cell level [31]. It can be combined with single-cell RNA sequencing (scRNA-seq) to measure the transcriptome and chromatin accessibility of the same single cell simultaneously [32].

Although chromatin accessibility can reflect TF binding to genomic DNA, to explore genome-wide TF–DNA interactions directly, chromatin immunoprecipitation followed by sequencing (ChIP-seq) was developed based on chromatin immunoprecipitation (ChIP), a technology developed in the 1980s for profiling interactions between specific proteins and chromatins [33–35]. Through ChIP, proteins are first covalently crosslinked to DNA *in vivo*. Next, the crosslinked chromatin is fragmented by sonication into small fragments of 200–600 bp, and the DNA–protein complexes of interest are enriched through immunoprecipitation using an immobilized target-specific antibody. After reversing the crosslinking, the DNA can be assessed to identify protein-binding sites in the genome. The DNA detection methods have evolved from an initial low-resolution approach and a labor-intensive Southern blot method to quantitative PCR, to subsequent DNA microarrays (ChIP-chip), and finally, to next-generation sequencing technology (ChIP-seq), which enables the genome-wide profiling of protein–DNA occupancy at single base-pair resolution [23,36] by directly sequencing the ends of the immunoprecipitated fragments (Figure 1C) [37–40]. Many studies quickly applied the ChIP-seq technique to detect DNA–protein interactions. Large genomics consortia, including the ENCODE [27], Model Organism Encyclopedia of DNA Elements (modENCODE) [41], Roadmap Epigenomics [42], and International Human Epigenome Consortium (IHEC) consortia [43], have generated tens of thousands of ChIP-seq data to profile the distribution of TFs, RNA polymerase, and histone modifications across entire genomes in various cell types in humans, mice, worms, and flies.

The ENCODE consortium established working guidelines and quality control (QC) standards to facilitate the success of the ChIP-seq assays [44]. However, ChIP-seq experiments must still be optimized based on the cell types, conditions, factors, and chromatin modifications being assayed due to various technical challenges [45]. Moreover, limitations are associated with conventional ChIP-seq, including the requirement for a large number of input cells (10^5^–10^7^ cells) due to the significant loss of cells during the immunoprecipitation step, time-consuming sample preparation protocols, potential false-positive results, and epitope masking due to the formaldehyde crosslinking process [46–48]. In 2017, Henikoff laboratory developed Cleavage Under Targets and Release Using Nuclease (CUT&RUN) followed by sequencing approach, which is a highly efficient method that uses a target-specific primary antibody and a protein A–protein G–micrococcal Nuclease (pAG–MNase) to digest and release specific protein–DNA complexes [49]. Building on the CUT&RUN protocol, in 2019, Kaya-Okur et al. [50,51] introduced the hyperactive Tn5 transposase. They developed Cleavage Under Targets and Tagmentation (CUT&Tag) followed by sequencing technology to overcome the major limitations of the conventional ChIP-seq method. No formaldehyde crosslinking or sonication-based fragmentation is needed. In contrast, CUT&Tag uses a specific antibody to bind the target protein or histone modification under native conditions. Subsequently, the system is introduced to a transposome composed of a hyperactive Tn5 transposase fused to a protein A (pA–Tn5), which has been preloaded with sequencing adapters. Tethering the transposome to the antibody and activating the Tn5 transposase with magnesium ions result in targeted DNA tagmentation, and these products can be directly amplified by PCR and sequenced (Figure 1C). For live cells, CUT&Tag provides amplified sequencing-ready libraries in a day without immunoprecipitation [51]. Another advantage of CUT&Tag is its higher sensitivity than ChIP-seq technology, which enables the detection of weak or low-affinity interactions that ChIP-seq may miss. The high activity and sensitivity of pA–Tn5 can effectively capture trace amounts of DNA with only a few binding sites in a small number of cells or even a single cell [52]. Additionally, CUT&Tag can be used to detect interactions that are difficult to study by ChIP-seq, such as those that exist in native conditions but could be very sensitive to the crosslinking step in the ChIP-seq protocol [51].

### Principles of data production and analysis and software introduction

In contrast to DNase-seq and ChIP-seq assays, in which DNA fragments are usually sequenced from only one end [single-end (SE) sequencing] after library construction, it is recommended that ATAC-seq and CUT&Tag libraries are sequenced in the paired-end (PE) mode [53]. PE mode sequencing can capture the two independent Tn5-guided primer insertions around target regions, eventually enabling better alignment efficiency for ATAC-seq and CUT&Tag libraries. Additionally, PE sequencing enables the after-alignment calculation of fragment length distribution, a crucial QC metric used to assess the efficiency of Tn5 fragmentation and the quality of DNA fragments used for ATAC-seq library construction. The fragment sizes obtained from ATAC-seq experiments can reveal periodic chromatin-associated patterns with a spatial frequency that aligns with nucleosomes, and a high-frequency periodic pattern corresponding to the helical pitch of the DNA in fragments shorter than 200 bp [30].

**Figure 2:**
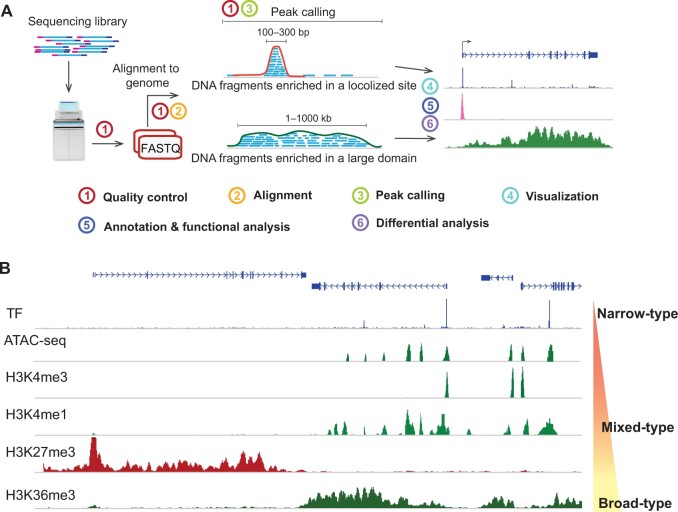
Data processing flow and different types of enriched epigenetic signals **A**. The principle underlying the analysis of ATAC-seq and CUT&Tag data. **B**. Browser view showing the narrow-type, mixed-type, and broad-type peaks in human IMR90 cells.

The principle underlying ATAC-seq and CUT&Tag data analyses generally involves the following major steps (**Figure 2**A). (1) QC. QC is an essential step in ATAC-seq and CUT&Tag data analyses because it helps ensure the analysis results’ accuracy, reliability, and validity. The standard of QC metrics of both ATAC-seq and CUT&Tag data have been studied extensively in previous reports [51,54–56]. QC of ATAC-seq and CUT&Tag data needs to be performed before alignment, after alignment, and after peak calling. Pre-alignment QC of a sequencing library is performed to evaluate the duplication rate, sequencing quality of reads, GC bias, library complexity, *etc*. After alignment, the unique mapping ratio, chromosome distribution, contamination, and insertion length distribution are examined to ensure sufficient quality data for downstream analysis. After peak calling, peak-associated QC is reviewed, including the reads under peak ratio, promoter enrichment, peak length distribution, and saturation. (2) Alignment. Sequencing reads are aligned/mapped to a reference genome. Short-read aligners like Bowtie and BWA can identify genomic locations where the reads originated based on the indexed genome reference. After alignment, only nonredundant, uniquely mapped reads are retained. These reads represent the true signals of DNA fragments in open chromatin regions or protein-binding regions, not potentially duplicated DNA fragments generated during the PCR amplification step. (3) Peak calling. Nonredundant, uniquely mapped reads will pile up on target regions along the genome and display a “peak” distribution compared to neighboring regions (Figure 2A). The procedure to identify these signal-enriched “peaks” is called “peak calling analysis”. Several tools, such as Model-based Analysis of ChIP-seq 2 (MACS2) and HMMRATAC, can directly use alignment results (in BAM format) to identify genomic regions with enriched ATAC-seq or CUT&Tag signals. Other tools, such as F-Seq, HOMER, and SEACR, can process only DNA fragment files in BED format. These tools are based on different peak calling algorithms or statistical methods and identify the genomic regions with significantly enriched signals compared to the background noise. (4) Visualization. Visualizing peak calling results and exploring relationships between identified peaks and nearby genes are important. Using tools such as BEDTools, bamCoverage, deepTools, and methylQA, alignment results (in BAM format) can be converted to bigWig or bedGraph format. Then, the DNA fragments of ATAC-seq or CUT&Tag data can be visualized. Most peak calling tools reveal the peaks in BED or bedGraph format. These BED, bedGraph, and bigWig files can be directly visualized with various genome browsers, such as the UCSC Genome Browser, Integrative Genomics Viewer (IGV), and the WashU Epigenome Browser. (5) Annotation and functional analysis. Identified peaks can be further annotated to indicate their genomic distribution (*e.g.*, their locations in the promoter, intron, and intergenic regions) and their relationships to nearby genes. HOMER is a software tool suite with peak calling, motif finding, and peak annotating functions. Other tools, such as GREAT, Cistrome-Go, ChIP-Atlas, and ChIP-Enrich, can directly use the genomic coordinates of peaks to perform functional analysis, including Gene Ontology analysis, pathway analysis, and gene set enrichment analysis, to explore further the biological functions enriched for the genes associated with peaks.

Depending on the target, the signal-enriched “peaks” form different shapes and can be classified into three types (Figure 2B). (1) In “narrow-type” peaks, signals are enriched at specific small regions in a genome. Most TFs, open chromatin regions, and active histone modifications (*e.g.*, H3K4me3 and H3K27ac), are associated with narrow-type peak enrichment of ATAC-seq or CUT&Tag/ChIP-seq signals. (2) In “broad-type” peaks, signals appear in large genome domains. Certain chromatin remodelers (*e.g.*, DNMT and MeCP2) and several histone modifications (*e.g.*, H3K36me3 and H3K9me3) are associated with broad-type peaks. (3) “Mixed-type” peaks represent a combination of narrow- and broad-type peaks, which are associated with, for example, RNA Polymerase II and repressive histone H3K27me3 mark signal enrichment.

Many bioinformatics tools have been developed to perform peak calling analysis for ChIP-seq assays, and most of them can be directly used to perform peak calling analysis of DNase-seq data. Since ATAC-seq and CUT&Tag data reveal signal distributions similar to those in ChIP-seq data, many of the tools developed for ChIP-seq data analysis can also be applied to ATAC-seq and CUT&Tag data. Although MACS2 is the best recognized peak calling analysis tool, other tools include SPP, F-Seq, and HOMER. Most of these tools first yield narrow regions with enriched signals compared to the global background distribution and neighboring regions and then merge nearby candidate regions to reveal wider final candidate regions. ChromHMM is a hidden Markov model-based method for learning and characterizing chromatin states. It can be used to analyze single or multiple ChIP-seq datasets and predict DNA modification status. Recently, tools have been developed specifically for analyzing ATAC-seq and CUT&Tag data. HMMRATAC, a hidden Markov model-based algorithm, was developed to analyze ATAC-seq data specifically. SEACR was developed to analyze CUT&RUN sequencing data [49]. To apply these tools, users must perform QC and complete the alignment, then feed the processed data (BAM/BED format files) to identify peaks. Many pipelines/packages have been developed to complete the peak calling analysis in a one-stop fashion to enable a more straightforward and convenient peak calling analysis. These packages integrate multiple bioinformatics tools in the required order to perform complete data analysis, including alignment, QC, peak calling, data visualization, and differential analysis using optimized parameters. For example, the ENCODE consortium developed different data processing pipelines for cross-consortium standardized “omics” data, including ChIP-seq, DNase-seq, and ATAC-seq data processing. AIAP is a recently published one-station pipeline that performs alignment, QC, peak calling, and differential analysis of ATAC-seq data with significantly increased sensitivity. CUT&RUNTools 2.0 was developed for interactive analysis of CUT&RUN and CUT&Tag data. Like AIAP, CUT&RUNTools 2.0 is used to align and perform peak calling analysis via the calling functions of MACS2 and SEACR. Here, we summarize the features of available peak callers in **Table 1**.

**Table 1:** Summary of tested bioinformatics tools for analyzing bulk ATAC-seq and CUT&Tag data

Software	Tested version (web site)	Data processing						
		Input data type	Trimming	Quality control	Mapping	Peak calling method	Visualization	Other features
AIAP	v1.1 (https://github.com/Zhang-lab/ATAC-seq_QC_analysis)	FASTQ	cutadapt	Yes	BWA	MACS2	BED, bigWig, bedGraph	Differential analysis, insertion free region discovery
HMMRATAC	v1.2.10 (https://github.com/LiuLabUB/HMMRATAC)	BAM	No	No	No	HMMRATAC	BED	No
MACS2	v2.2.7.1 (https://github.com/macs3-project/MACS)	BAM/BED	No	No	No	MACS2	BED	No
SEACR	v1.3 (https://github.com/FredHutch/SEACR)	bedGraph	No	No	No	SEACR	BED	No
ChromHMM	v1.23 (https://ernstlab.biolchem.ucla.edu/ChromHMM/)	BED/BAM	No	No	No	ChromHMM	BED	No
CUT&RUNTools 2.0	Version published on 09/02/2021 (https://github.com/fl-yu/CUT-RUNTools-2.0)	FASTQ	Trimmomatic	No	Bowtie2	MACS2, SEACR	BED	Single-cell analysis
F-Seq	v1.85 (https://fureylab.web.unc.edu/software/fseq/)	BED	No	No	No	F-Seq	BED, bigWig	No
HOMER	v4.11 (http://homer.ucsd.edu/homer/index.html)	BAM	No	No	No	HOMER	BED	Motif analysis, genomic annotation, Gene Ontology enrichment

## Results

### Performance comparison of bioinformatics tools for ATAC-seq data analysis

**Figure 3:**
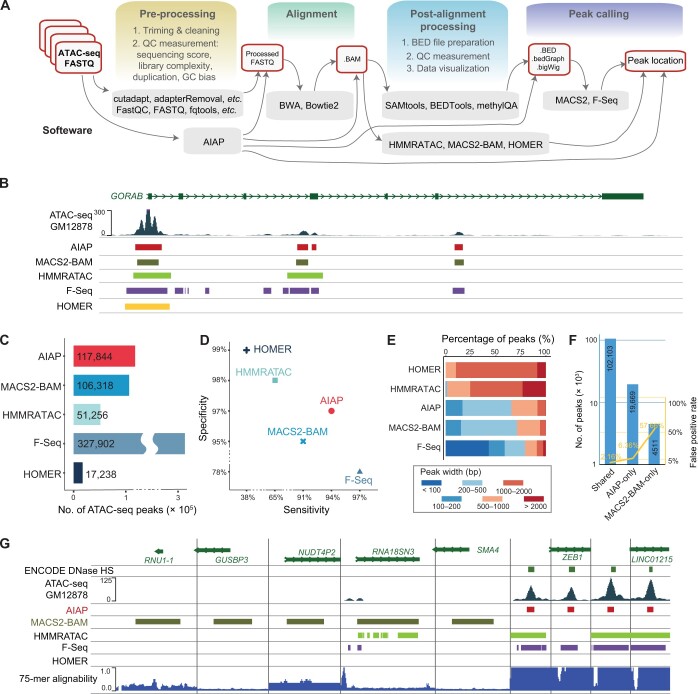
Performance comparison of bioinformatics tools used in ATAC-seq data analysis **A**. ATAC-seq data processing flow chart. **B**. Browser view of peak calling results for human GM12878 cells. **C**. The number of ATAC-seq peaks identified by the five bioinformatics tools. **D**. Sensitivity and specificity of the five bioinformatics tools for identifying ATAC-seq peaks. **E**. Peak width distribution of the ATAC-seq peaks identified by the five bioinformatics tools. **F**. False positive rate (yellow line) of the ATAC-seq peaks (shared and method-specific) identified by AIAP and MACS2-BAM. **G**. Browser view of the false-positive ATAC-seq peaks identified only by MACS2-BAM (five examples on the left side) and true-positive ATAC-seq peaks identified only by AIAP (four examples on the right side). QC, quality control; MACS2, Model-based Analysis of ChIP-seq 2; HS, hypersensitive site.

First, ATAC-seq data need to be aligned to a genome, and QC metrics need to be examined (**Figure 3**A). To identify the best strategy for ATAC-seq data pre-processing and peak calling, we evaluated the performance of five bioinformatics tools, namely, AIAP, F-Seq, HMMRATAC, HOMER, and MACS2. AIAP can perform complete data analysis, starting from the raw data in FASTQ format and producing peak calling results with a comprehensive QC metric report. HOMER, HMMRATAC, and MACS2 can perform data analysis, starting with the alignment results in BAM format generated by BWA or Bowtie2, and producing peak calling results but no QC report. F-Seq and MACS2 can be used to perform data analysis starting with processed DNA fragment files in BED format generated by further processing of BAM alignment files using SAMtools, BEDTools, or methylQA and producing peak calling results. Considering the different characteristics of these tools, we designed a benchmarking flow chart (Figure S1) following the data processing procedures illustrated in Figure 3A. We chose the Omni-ATAC data of GM12878 human cells (SRX2717911 dataset) [57], which was processed by using AIAP to generate a QC report (Table S1), peak calling results, an alignment file (in BAM format), and a processed DNA fragment file (in BED format). The BAM file was then used as the input file for HOMER, HMMRATAC, and MACS2 peak calling for the GM12878 data. The BED file was the input file for the F-Seq peak calling for the GM12878 data. All the peak calling results of the five tools (Figure 3B) were compared to the DHSs reported by ENCODE, an independent dataset of open chromatin regions identified in GM12878, to evaluate tool sensitivity and specificity.

We noticed that these five tools generated different numbers of peaks from the same ATAC-seq data (Figure 3C). F-Seq reported the highest number of peaks (327,902), and HOMER reported the lowest number of peaks (17,238). HMMRATAC, MACS2-BAM, and AIAP identified 51,256, 106,318, and 117,844 peaks, respectively. Based on the ENCODE DHS reference data (Figure 3D), F-Seq showed the highest sensitivity (97%) but the lowest specificity (78%). In contrast, HOMER showed the lowest sensitivity (38%) and the highest specificity (99%), followed by HMMRATAC (sensitivity: 65%; specificity: 98%). AIAP and MACS2-BAM showed a good balance between sensitivity (AIAP: 94%; MACS2: 91%) and specificity (AIAP: 97%; MACS2: 95%), with AIAP showing better performance than MACS2-BAM in this test. Most open chromatin regions identified by ATAC-seq can be considered candidate *cis*-regulatory elements (cCREs), and the average length of the cCREs discovered by ENCODE was ∼ 270 bp [58]. We calculated the peak width distribution to understand the size of the open chromatin regions reported by each tool evaluated (Figure 3E). Approximately 85% of the peaks identified by HOMER and 75% identified by HMMRATAC were longer than 1000 bp. In contrast, ∼ 40% of peaks identified by F-Seq were shorter than 100 bp. The ATAC-seq peaks identified by AIAP and MACS2-BAM exhibited a similar size distribution: ∼ 50% were in the size range of 200–500 bp. We noticed that AIAP performed comparably to MACS2-BAM with a slight advantage in peak number, sensitivity, and specificity. We further explored the difference in results obtained with AIAP and MACS2-BAM, since AIAP is based on the MACS2 peak calling function but includes an optimized data preparation function [55]. Compared to MACS2-BAM, AIAP identified ∼ 20% more peaks, and ∼ 94% of these peaks were validated by comparison with the DHSs. In addition, 4511 peaks were identified only by MACS2-BAM. However, ∼ 57.66% of these peaks were located outside the DHSs and can be considered false positives (Figure 3F). After examining some false positive peaks identified by MACS2-BAM, we noticed that many of these peaks were located in regions with low mappability (Figure 3G), suggesting that MACS2-BAM might include multiple mapped reads located in the low mappable areas in the peak calling process. In general, our comparison of the ATAC-seq data analysis results indicates that AIAP performs better in peak calling with balanced sensitivity and specificity. In contrast to the other tools evaluated, AIAP also produces detailed QC reports and visualization files that can be directly accessed in genome browsers.

### Performance comparison of bioinformatics tools for CUT&Tag data analysis

**Figure 4:**
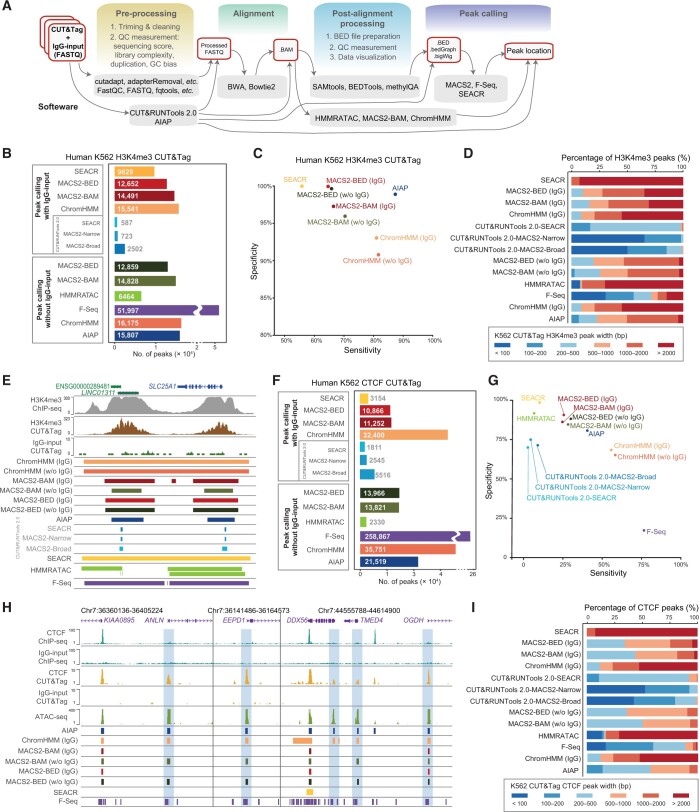
Performance comparison of bioinformatics tools in the peak calling analysis of narrow-type CUT&Tag data **A**. Narrow-type CUT&Tag data processing flow chart. **B**. Number of H3K4me3 CUT&Tag peaks in human K562 cells identified by the tested bioinformatics tools. **C**. Sensitivity and specificity of the tested bioinformatics tools for identifying H3K4me3 CUT&Tag peaks. **D**. Peak width distribution of the H3K4me3 CUT&Tag peaks identified by the tested bioinformatics tools. **E**. Browser view of K562 human cell H3K4me3 CUT&Tag peak calling results. **F**. Number of CTCF CUT&Tag peaks identified by the tested bioinformatics tools in human K562 cells. **G**. Sensitivity and specificity of the tested bioinformatics tools for identifying CTCF CUT&Tag peaks. **H**. Browser view of potential false-positive CTCF CUT&Tag peaks (light blue) overlapping with the ATAC-seq signals but not overlapping with the CTCF ChIP-seq signals. **I**. Peak width distribution of CTCF CUT&Tag peaks identified by the tested bioinformatics tools.

The recently developed CUT&Tag is an alternative to the classic ChIP-seq method. The highly efficient tagmentation and ligation properties of Tn5 enable the simplification of experimental procedures and reduce the control IgG input requirements for the starting cell population, with even single-cell input as an option. These unique advantages quickly led to the extensive application of CUT&Tag to different kinds of epigenetic profiling analysis. However, the optimal data processing strategy for CUT&Tag data has not yet been thoroughly investigated. In most cases, users use the tools developed for analyzing ChIP-seq or CUT&RUN data, such as MACS2 and SEACR, to process CUT&Tag data. Here, we performed a comprehensive comparison to evaluate the performance of available bioinformatics tools, including AIAP, ChromHMM, CUT&RUNtools 2.0, F-Seq, HMMRATAC, MACS2, and SEACR, for analyzing CUT&Tag data. In contrast to those used with ATAC-seq technology, it is recommended that IgG control data be used with the CUT&Tag method to reduce the number of potential false positives caused by antibody aggregation. However, we noticed that in many CUT&Tag datasets, genomic DNA input was used as the control or no control IgG-input data were used. Therefore, we designed two analyses to account for these real-life situations. (1) We evaluated the performance of AIAP, ChromHMM, F-Seq, HMMRATAC, and MACS2 in processing CUT&Tag data without control IgG-input data. (2) We also analyzed the performance of CUT&RUNtools 2.0, ChromHMM, MACS2, and SEACR in processing CUT&Tag data with corresponding control IgG-input data (Figure S1). We analyzed MACS2 performance by using either alignment data (in BAM format, MACS2-BAM) or processed alignment data (in BED format, MACS2-BED) as the input file for peak calling (**Figure 4**A). In our testing, we performed such analyses with both narrow-type and broad-type peak data.

### Peak calling analysis of narrow-type CUT&Tag data

The active histone modifications of genomic DNA and most TFs that bind to the genome are generally limited to a localized region, resulting in the narrow-type peaks of signal enrichment based on data obtained from the ChIP-seq and CUT&Tag methods [50,51]. To evaluate the performance of all the bioinformatics tools in analyzing narrow-type CUT&Tag peak data, we first analyzed the active histone modification H3K4me3 in the CUT&Tag data (GSM3536518, 25 bp, PE) from the human K562 cell line generated by the Henikoff laboratory (Figure 4B). ChromHMM and MACS2-BAM reported a similar number of peaks: 15,541 and 14,491 for analyses with control IgG-input data. MACS2-BED generated 12,652 H3K4me3 peaks, and SEACR reported 9629 peaks. In this analysis, CUT&RUNTools 2.0 identified very few H3K4me3 peaks either in MACS2 peak calling mode or SEACR peak calling mode, even after changing different condition parameters. In analyzing data without control IgG-input data, the most significant number of peaks were identified by F-Seq (51,997), and HMMRATAC identified the least number of peaks (6464). AIAP reported 15,807 H3K4me3 peaks without control IgG-input data. ChromHMM, MACS2-BAM, and MACS2-BED reported similar numbers of peaks as those identified by peak calling with control IgG-input data, suggesting that the loss of control IgG-input data do not affect the peak calling efficiency of these tools.

Next, we used human K562 H3K4me3 ChIP-seq data from the ENCODE database as a reference dataset to evaluate the sensitivity and specificity of all the tools in analyzing H3K4me3 CUT&Tag data (Figure 4C). When control IgG-input data were included for the peak calling analysis, ChromHMM showed the highest sensitivity (81%) but lower specificity (93%) among all the tested tools. MACS2-BED and SEACR showed the highest specificity, close to 100%, but MACS2-BED showed higher sensitivity (64%) since it identified ∼ 31% more peaks than SEACR. MACS2-BAM showed slightly better sensitivity (66%) than MACS2-BED but lower specificity (97%). Because of the low number of identified peaks, CUT&RUNTools 2.0 showed relatively low sensitivity (< 13%) in this analysis. A peak calling analysis of H3K4me3 CUT&Tag data without control IgG-input data was performed for comparison. Generally, without control IgG-input data, the specificity of the tools was reduced, but the sensitivity was increased, as expected. The performance of MACS2-BED was largely unaffected when the control IgG-input data were not included. At the same time, MACS2-BAM showed a ∼ 2% loss in specificity with a 4% increase in sensitivity when control IgG-input data were not included. ChromHMM showed a ∼ 2% loss in specificity with a 1% increase in sensitivity when the control IgG-input data were not included. Interestingly, AIAP showed high specificity (∼ 99%) and the highest sensitivity of 88% among all the tested tools.

We also evaluated the width distribution of H3K4me3 peaks identified by different tools (Figure 4D and E). CUT&RUNTools 2.0 and F-Seq reported relatively narrow peaks (< 500 bp), while SEACR, HMMRATAC, and ChromHMM generated much broader H3K4me3 peaks (> 2000 bp). We also evaluated the performance of these tools in analyzing CUT&Tag data for another active histone modification, H3K27ac, and the general performance of each tool was similar to its performance in analyzing H3K4me3 CUT&Tag data (Figure S2A). We also analyzed tool performance with another mouse embryonic stem cell (mESC) H3k4me3 CUT&Tag dataset (100 bp, PE), and we found that the performance of CUT&RUNTools 2.0 was markedly increased and was comparable to that of MASC2 and ChromHMM (Figure S2B).

To evaluate the sensitivity and specificity of bioinformatics tools in analyzing TF CUT&Tag data, we used CTCF CUT&Tag data from the human K562 cell line generated by the Henikoff laboratory (Figure 4F) and CTCF ChIP-seq data from ENCODE as the reference (see Materials and methods). We performed an analysis similar to that with the H3K4me3 CUT&Tag data, with or without control IgG-input data. Overall, the performance of each tool in analyzing CTCF CUT&Tag data was similar to its performance in analyzing H3K4me3 CUT&Tag data (Figure 4G). However, we noticed that all the tools showed relatively low sensitivity in analyzing the CTCF CUT&Tag data: sensitivity for F-Seq and AIAP reached only 76% and 38%, respectively (Figure 4G). We further explored the CTCF peaks produced by AIAP and found that 17.3% of the CTCF CUT&Tag peaks overlapped with open chromatin regions but not with CTCF ChIP-seq peaks, and 100% of these CTCF peaks were free of CTCF-binding motifs (Figure 4H, Figure S2C). These results suggest that CTCF CUT&Tag data might capture certain levels of open chromatin regions or indirect binding events. CTCF might work as a cofactor but not directly bind to genomic DNA. We finally evaluated the CTCF peak width distribution. SEACR, ChromHMM, and HMMRATC produced broader peaks, and the distributions of these peaks were similar to those of the H3K4me3 peaks (Figure 4I). CTCF peaks generated by CUT&RUNTools 2.0 were much narrower, and their distributions were similar to those of the H3K4me3 peaks called by CUT&RUNTools 2.0 (Figure 4I). AIAP, F-Seq, and MACS2 produced narrower CTCF peaks than their H3K4me3 peaks.

### Peak calling analysis of broad-type CUT&Tag data

**Figure 5:**
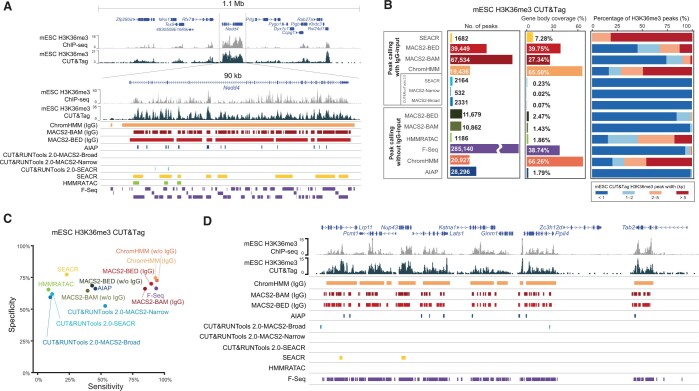
Performance comparison of bioinformatics tools in the peak calling analysis of broad-type CUT&Tag data **A**. Browser view of H3K36me3 CUT&Tag peak calling results in mESCs. **B**. Left: number of H3K36me3 CUT&Tag peaks identified by the tested bioinformatics tools. Middle: gene body coverage of the H3K36me3 peaks identified by the tested bioinformatics tools. Right: peak width distribution of the H3K36me3 peaks. **C**. Sensitivity and specificity of the bioinformatic tools used to identify H3K36me3 CUT&Tag peaks. **D**. Browser view of H3K36me3 CUT&Tag peaks identified only by ChromHMM, MACS2, and F-Seq. mESC, mouse embryonic stem cell.

Compared to the ChIP-seq method, the high sensitivity and low background noise of the CUT&Tag method enable it to be extensively used to measure broad-type histone modifications. To evaluate the performance of the bioinformatics tools in analyzing broad-type CUT&Tag data, H3K36me3 CUT&Tag data of mESCs were used as the benchmarking data. The trimethylation of the 36th lysine residue in the histone H3 protein (H3K36me3) is enriched mainly in the gene bodies and is often associated with the serine-2-phosphorylated elongating form of RNA polymerase II [17,18] (**Figure 5**A). After alignment, we calculated the accumulated H3K36me3 signals at each gene based on the coordinates of the transcription start sites and transcription termination sites. Then, 10% of genes with the highest enrichment of H3K36me3 signals at the gene body were used as the reference genes to evaluate the peak calling performance of different bioinformatics tools (referred to as sensitivity gene set). On top of the selected sensitivity gene set, the genes with accumulated H3K36me3 signals at the gene body [reads per kilobase per million mapped reads (RPKM) > 0.5] were used as the specificity reference gene set to determine whether the peaks called by different bioinformatics tools were true or false peaks.

Similar to the previous analysis, F-Seq identified the greatest number of H3K36me3 peaks among all the tools, followed by MACS2-BAM (IgG) and MACS2-BED (IgG) (Figure 5B). The performance of MACS2 peak calling without control IgG-input data was markedly reduced, but that of ChromHMM was insensitive to the availability of control IgG-input data in the peak calling process. Since H3K36me3 modifications are usually located within a gene body, we calculated the gene body coverage of the top 10% of H3K36me3-enriched genes (the sensitivity gene set) by H3K36me3 peaks identified by different tools. Peaks identified by ChromHMM showed the highest coverage (> 65%) of the gene body in the sensitivity gene set, followed by MACS2-BAM (IgG), MACS2-BED (IgG), and F-Seq (Figure 5B). Other tools failed to cover more than 10% of a gene body in the sensitivity gene set. The H3K36me3 peak width distribution of all the tools was similar to previous analyses, with SEACR, ChromHMM, and HMMRATAC tending to report broader peaks. In the sensitivity–specificity analysis, no tools reached 80% specificity, suggesting that over 20% of the peaks identified by all tools could be located in intergenic regions, which are not included in the reference gene bodies (Figure 5C). ChromHMM and F-Seq identified > 90% of the genes in the sensitivity gene set. Including control IgG-input data in peak calling markedly reduced the sensitivity of MCAS2, as indicated by the number of identified peaks. Overall, ChromHMM performed better in identifying H3K36me3 broad-type peaks, showing a good balance among peak number, specificity, and gene body coverage. When evaluating the identified peaks in the genome browser (Figure 5D), single ChromHMM peaks covered the regions enriched with H3K36me3 signals. This pattern of large peaks identified by ChromHMM reveals high sensitivity for downstream differential analysis.

### Differential peak analysis of CUT&Tag data

**Figure 6:**
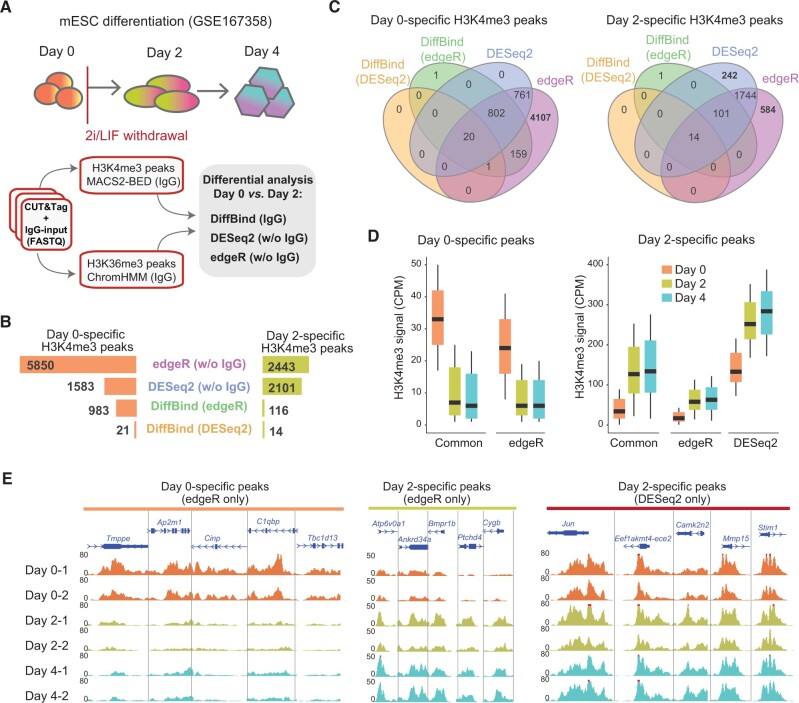
Performance comparison of bioinformatics tools in the differential analysis of CUT&Tag data **A**. Design of the differential analysis for evaluating mESC differentiation using bioinformatics tools. **B**. Number of differential H3K4me3 peaks identified by edgeR, DESeq2, and DiffBind. **C**. The intersection of the analysis results among edgeR, DESeq2, and DiffBind. **D**. H3K4me3 signal density on differential H3K4me3 peaks during mESC differentiation. **E**. Browser view of H3K4me3 CUT&Tag peaks identified only by edgeR (left and middle) and DESesq2 (right). CPM, counts per million.

After identifying enriched peaks, the ATAC-seq and CUT&Tag data can be used for differential peak analysis to explore dynamic changes in chromatin accessibility, histone modification, and TF binding. Dynamic changes in these factors on the epigenetic landscape are generally closely associated with gene regulation. Inspired by a previous benchmarking study showing a differential analysis of ATAC-seq data [59], we examined a similar differential analysis strategy with CUT&Tag data obtained from a mESC differentiation model [60]. Histone modifications were measured at three time points (Day 0, Day 2, and Day 4) after mESCs were induced to differentiate, and dynamic changes in the epigenetic landscape were analyzed using CUT&Tag (**Figure 6**A). Considering the benchmarking results described above, we chose MACS2-BED (IgG) to perform the peak calling analysis of the H3K4me3 CUT&Tag data. After peak calling, we chose three of the most widely used bioinformatics tools to perform a differential analysis of the CUT&Tag data measured on Day 0 and Day 2: (1) DiffBind was used to identify differentially methylated peaks (DMPs) between these two time points by considering the control IgG-input data; (2) DESeq2 and edgeR were used to identify DMPs without considering the control IgG-input data (Figure 6A). Since DiffBind can call differential peaks from the DESeq2 or edgeR package separately during the differential analysis, we were able to explore the importance of control IgG-input data in this differential analysis. We evaluated signal enrichment in samples on Day 4 to validate the results. Along with the mESC differentiation, the Day 0-specific peaks should remain at lower signals on Day 4, and Day 2-specific peaks should maintain higher signals on Day 4.

Without considering the control IgG-input data during the analysis, edgeR (w/o IgG) identified the greatest number of DMPs, followed by DESeq2 (Figure 6B). After combining the control IgG-input data information, DiffBind identified only a limited number of DMPs between Day 0 and Day 2, and DiffBind-edgeR performed better than DiffBind-DESeq2. We noticed that > 99% of DMPs identified by DiffBind were all identified by edgeR, and all the DESeq2-identified Day 0-specific H3K4me3 DMPs were identified by edgeR. DESeq2 identified 242 Day 2-specific H3K4me3 DMPs which were not identified by edgeR (Figure 6C). To evaluate the quality of DMPs identified by DESeq2 and edgeR, we further explored the H3K4me3 signals on the identified DMPs on Day 0, Day 2, and Day 4. Results suggest the high quality and confidence of the DMPs identified by both DESeq2 and edgeR: on Day 4, Day 0-specific H3K4me3DMPs maintained low signals, and Day 2-specific H3K4me3DMPs maintained higher signals, as expected (Figure 6D). We also noticed that DMPs specifically identified by DESeq2 showed an overall higher H3K4me3 signal [counts per million (CPM)] than edgeR-specific DMPs, suggesting the potential difference in data normalization and differential modeling (Figure 6D and E).

## Discussion

Epigenetic regulation bridges gene expression and the external environment and plays determinative roles during embryo development and disease genesis. Tn5-based experimental approaches, including ATAC-seq and CUT&Tag, are straightforward methodologies to explore chromatin accessibility, histone medication, and protein–DNA interactions with high efficiency and sensitivity. Along with simplified experimental protocols and decreased sequencing costs, an increasing amount of epigenomic data have been generated acceleratedly. Appropriately analyzing these rapidly generated data has become a bottleneck in recent research. Here, we reviewed the development and application of ATAC-seq and CUT&Tag methods, both taking advantage of the high-efficiency tagmentation property of Tn5 to generate high-quality data with low background noise. ATAC-seq and CUT&Tag data showed similar signal distributions to traditional DNase-seq and ChIP-seq data. These characteristics allowed us to apply ChIP-seq data analysis strategies directly to ATAC-seq and CUT&Tag data analyses.

We reviewed the principles underlying the data analysis programs and evaluated the performance of the bioinformatics tools most widely used for ATAC-seq data analysis. Our analysis revealed distinctive difference in the performance of different tools in terms of peak calling sensitivity and specificity, as well as peak width distribution. For ATAC-seq analysis, among the five evaluated bioinformatics tools, AIAP showed a balanced performance in terms of peak number identification, sensitivity, specificity, and peak width. AIAP processed the PE ATAC-seq data into independent SE Tn5 insertion events and then adopted MACS2 to perform peak calling. This modified data processing strategy allowed AIAP to identify ∼ 17% more peaks with 94% specificity than those identified with MACS2, the tool showing the second-best performance. AIAP also simultaneously generated a report with comprehensive QC measurement metrics.

The highly efficient tagmentation and low background noise of the CUT&Tag method enabled it to quickly replace the traditional ChIP-seq method and to be widely used for many biomedical studies. The unique Tn5 reactions in native (non-crosslinked) nuclei enable the CUT&Tag method to be applied to a small number of input nuclei, even at the single-nucleus level [51]. In contrast to ATAC-seq technology, generating a parallel control IgG-input dataset is recommended for the CUT&Tag protocol to evaluate non-specific antibody enrichment and control the overall background. However, sometimes, due to the limited availability of input cell numbers such as clinical samples, control IgG-input data are not available. An increasing number of researchers are choosing to omit the step required to generate control IgG-input data. Therefore, our evaluation of the performance of popular bioinformatics tools for peak calling using CUT&Tag data with or without control IgG-input data provided valuable information to the research community. We noticed that whether or not to include control IgG-input data in the analysis differentially affects the peak calling performance depending on the peak type.

MACS2, AIAP, and ChromHMM showed high sensitivity and specificity in narrow-type peak calling analysis. SEACR showed the highest specificity among all the peak calling programs, but its overall sensitivity was relatively low when the parameters were set to default. In contrast to MACS2 and ChromHMM, for which a statistical cutoff is applied to fine-tune the enrichment calculation, the SEACR default setting is based on empirical filtering considering control IgG-input data. Alternatively, users can set the percentile cutoff to select the most enriched regions, leading to generating a much higher number of identified peaks. To our surprise, CUT&RUNTools 2.0 showed very unsatisfactory performance in our analysis. This outcome might have been caused by the short-read length (25 bp PE) in the H3k4me3 and CTCF CUT&Tag data. When used for analyzing mESC H3k4me3 CUT&Tag dataset (100 bp PE), CUT&RUNtools 2.0 showed markedly improved performance, which became comparable to that of MASC2 and ChromHMM (Figure S2B). The performance of AIAP revealed a good balance between sensitivity and specificity, suggesting that optimized ATAC-seq data processing can be used for a better CUT&Tag data analysis.

When considering the effect of including control IgG-input data during peak calling, MACS2 (using BAM or BED files as input files) and ChromHMM showed higher specificity but loss of sensitivity. In the H3K4me3 peak calling analysis, the performance of MACS2-BED was almost the same with or without control IgG-input data. Therefore, the recommendation to include control IgG-input data for narrow-type peak calling of CUT&Tag data should be revisited. In contrast to ChIP-seq data, which tend to show more background noise but fewer false positives, CUT&Tag might show bias in the other direction: low background noise but a higher rate attributed to open chromatin regions and indirect biding events, and such differences were also reported in CUT&RUN experiments before [49]. However, such differences in the same TF between ChIP-seq data and CUT&Tag data could be associated with many factors, including the difference in antibodies, sample processing, library construction, and sequencing platform. Thus, one limitation of our current benchmarking is that we could not systematically explore these confounding factors because of the missing information. We also noticed that different tools generated different peak width distributions. F-Seq, MACS2, AIAP, and CUT&RUNtools 2.0 tended to identify narrower regions as peaks (< 1 kb), whereas SEACR, ChromHMM, and HMMRATAC usually identified much wider peak regions (> 2 kb). The broader peaks might increase the difficulty of downstream TF-binding motif enrichment analysis. Users should carefully choose peak callers or fine-tune the peak calling results to facilitate downstream analysis.

In contrast to its effect on narrow-type peak calling, control IgG-input data seemed to exert a marked impact on broad-type peak calling. With control IgG-input data, the sensitivity of MACS2 increased by more than 40% in our analysis. However, even in the broad-type peak calling mode, MACS2 tended to identify narrow peaks (< 2 kb) for the widely distributed histone modification H3K36me3, which usually spans a whole gene body. ChromHMM was not sensitive to the lack of IgG-input data and showed better sensitivity, specificity, and whole gene body coverage. This performance confers extra advantages to subsequent downstream differential analysis because the H3K36me3 peaks identified by ChromHMM can be easily assigned to genes in a one-to-one relationship, which increases the statistical power by increasing the signal density and reducing the number of tests required for multiple testing correction.

In our differential analysis, control IgG-input data markedly affected the identification efficiency of DMPs related to H3K4me3 and H3K36me3 marks. DiffBind with control IgG-input data in differential analysis identified a limited number of DMPs. If control IgG input was not included in the analysis of CUT&Tag data of histone methylation, DESeq2 and edgeR reported much larger DMPs. edgeR seemed to show higher sensitivity in low-signal regions, but DESeq2 identified more relatively high-signal regions. Overall, edgeR showed a better performance in our analysis, and the Day 0- and Day 2-specific DMPs identified by edgeR were validated through a biological analysis with a dataset obtained later in the experiment (Day 4).

However, our testing had certain limitations. (1) The sensitivity and specificity calculation for all the benchmarking relays on the reference dataset. To ensure a fair comparison, we chose the independent ENCODE DHSs as reference standards for ATAC-seq data testing. ChIP-seq peak calling results from the ENCODE consortium, including H3K4me3, H3K27ac, and CTCF, were used as independent reference standards for CUT&Tag data testing. Specifically, we chose expressed genes with the highest enrichment (top 10%) of H3K36me3 signals in their gene bodies for the broad-type peak calling as reference standards for H3K36me3 CUT&Tag data testing. These reference datasets could be better, but the independence of such reference datasets ensured the unbiased evaluation of all the tools in the benchmarking. (2) The peak width distributions and peak numbers of all the peak calling software are mainly determined by the model and initial parameters designed by the developers. In our testing, we adopted most of the default parameters for the tools and benchmarked the final output unbiasedly. The users might obtain better performance by optimizing the specific parameters of tools. (3) During the benchmarking, we only tested the data generated from human and mouse cell lines; tools’ performance might differ in other organisms or specific tissue types.

In summary, we reviewed the development and application of ATAC-seq and CUT&Tag technologies and performed comprehensive benchmarking analysis using different types of true biological data generated with these technologies. For ATAC-seq analysis, we recommend AIAP, which showed better performance in generating high-quality peaks and simultaneously generated comprehensive QC reports. AIAP can be used to analyze raw FASTQ files initially and produce the peak calling results, visualization files, and QC reports using a single command. For narrow-type peak CUT&Tag data analysis, MACS2-BED is highly recommended when control IgG-input data are available; however, AIAP is recommended when control IgG-input data are unavailable. For broad-type peak CUT&Tag data analysis, we recommended ChromHMM, which was insensitive to the control IgG-input data, to define large domains with histone modification marks. For the subsequent downstream differential analysis, we recommend using edgeR to obtain results with high sensitivity, and we recommend DiffBind(edgeR) when high specificity is needed and control IgG-input data are available.

## Materials and methods

### Terminology definition

Sensitivity: the proportion of the reference peaks which are considered as gold-standard true-positive peaks that overlap with the peaks identified by tested software.

(1) $$\mathrm{Sensitivity}=\frac{\mathrm{Number}\;\mathrm{of}\;\mathrm{overlapping}\;\mathrm{peaks}\;\mathrm{called}\;\mathrm{by}\;\mathrm{tested}\;\mathrm{software}}{\mathrm{Number}\;\mathrm{of}\;\mathrm{total}\;\mathrm{peaks}\;\mathrm{of}\;\mathrm{the}\;\mathrm{sensitivity}\;\mathrm{reference}\;\mathrm{dataset}}\times 100\%$$

Specificity: the proportion of peaks identified by tested software that overlap with reference peaks which are considered as the gold-standard true-positive peaks.

(2) $$\mathrm{Specificity}=\frac{\mathrm{Number}\;\mathrm{of}\;\mathrm{overlapping}\;\mathrm{peaks}\;\mathrm{called}\;\mathrm{by}\;\mathrm{tested}\;\mathrm{software}}{\mathrm{Number}\;\mathrm{of}\;\mathrm{total}\;\mathrm{peaks}\;\mathrm{called}\;\mathrm{by}\;\mathrm{tested}\;\mathrm{software}}\times 100\%$$

Gene body coverage: the proportion of reference gene bodies (true positives) overlapped by the H3K36me3 peaks identified by tested software.

(3) $$\begin{array}{c} \mathrm{Gene}\;\mathrm{body}\;\mathrm{coverage}=\\ \frac{\begin{array}{c} \mathrm{Length}\;\left(\mathrm{bp}\right)\;\mathrm{of}\;\mathrm{reference}\;\mathrm{gene}\;\mathrm{bodies}\;\mathrm{overlapped}\;\mathrm{by}\;\mathrm{identified}\;\mathrm{peaks} \end{array}}{\mathrm{Total}\;\mathrm{length}\;\left(\mathrm{bp}\right)\;\mathrm{of}\;\mathrm{reference}\;\mathrm{gene}\;\mathrm{bodies}}\times 100\% \end{array}$$

### Benchmarking ATAC-seq peak calling software

#### Data used in the study

The data used in this study are listed in Table S1. ATAC-seq data of GM12878 cells generated by the Greenleaf laboratory with the Omni-ATAC protocol [57] was downloaded from the Gene Expression Omnibus (GEO) database (GSM1155957) as the test data for benchmarking ATAC-seq peak calling software. Known GM12878 DHSs (ENCSR000EMT) [61] obtained from the ENCODE data portal were used as the gold-standard true-positive reference data to validate the ATAC-seq peaks detected by all five software. Complete DHSs of 733 human biosamples (ENCFF503GCK) were used to measure the specificity of identified ATAC-seq peaks [62].

#### AIAP

AIAP [55] (v1.1 Singularity image, https://github.com/Zhang-lab/ATAC-seq_QC_analysis) was used with default setting following the instructions (genome assembly as hg38, read type as PE). AIAP consists of four steps: data processing, QC, integrative analysis, and data visualization. (1) Data processing. PE raw reads are trimmed by cutadapt [63] and aligned to the reference genome by BWA [64], and the resulting alignment BAM-format files are processed by methylQA in the ATAC mode. (2) QC. AIAP performs multiple steps of quality checking before and after read alignment, such as reports of sequencing quality, duplication rate, GC bias by FastQC before alignment, and mapping statistic summary, chromosome distribution of uniquely mapped reads, and peak width distribution after alignment. (3) Data visualization. It generates a suite of data visualization files that can be directly used as input files to a genome browser [65], including bigWig-format normalized signal density files, Tn5 insertion position files, BED-format peak files, and footprint position files. It also generates JSON-format QC report files that can be visualized via an embedded qATACviewer [55].

#### HMMRATAC

HMMRATAC [66] (https://github.com/LiuLabUB/HMMRATAC) will first decompose the ATAC-seq dataset into different layers of coverage signals, which is based on expectation maximization (EM) algorithm for Gaussian mixture models. The relationships between the layers of signals at open chromatin regions are learned in a hidden Markov model and refined with the Baum–Welch algorithm, which are then utilized for predicting accessible regions across the whole genome by the model. HMMRATAC v1.2.10 was used to call peaks using the trimmed PE BAM files created by AIAP. HMMRATAC was run with the “-u” option as 35 and the “-l” option as 2 to increase the number of peaks. We also added “--bedgraph true” option to facilitate the downstream analysis and visualization.

#### MACS2

MACS2 [67] (https://github.com/macs3-project/MACS) is the most widely used peak calling software for ChIP-seq data. MACS2 v2.2.7.1 was used to call peaks using the trimmed BAM files created by AIAP as the input files. During each run, the input file format (option “-f”) was set to “BAM”, and the option for keeping duplicate reads (option “--keep-dup”) was set to “1000”. MACS2 was run with shifting model pattern (option “—nomodel”) and the *q*-value (option “-qvalue”) was set equal to 0.01. We used the default value of shift option (option “--shift”), and the size of binding regions for ATAC-seq TF (option “--extsize”) was set to 150.

#### F-Seq

F-Seq [68] (https://fureylab.web.unc.edu/software/fseq/) is a software suite that produces a continuous estimation of tag sequence density, enabling the discernment of significant biological features, including TF-binding locations in ChIP-seq or areas of open chromatin in DNase-seq. Its features include automatically estimated fragment length, strict enforcement of strands, and the background model. F-Seq v1.85 was used to call peaks using the BED files generated by AIAP as the input files. The output file format was set to BED file (option “-of”), and the standard deviation cutoff was set to 4 (default).

#### HOMER

HOMER [69] (http://homer.ucsd.edu/homer/index.html) is a suite of tools for motif discovery and next-generation sequencing analysis. HOMER v4.11 was used to find enriched peaks by findPeaks program contained in HOMER with the minimum peak width set as 150 (option “-minDist”) and the option “-region” was used to find peaks that are more conforming to the regions of enrichment. Before finding peaks, makeTagDirectory, which is another program contained in HOMER, was used to make a HOMER tag directory from the BAM files of mapped ATAC-seq reads, which was generated by AIAP. Finally, the HOMER peak file was converted to a merged, sorted BED file by program pos2bed.pl in the HOMER suite.

### Benchmarking CUT&Tag peak calling software

#### Data used in the study

The data used in this study are listed in Table S1. The H3K4me3, CTCF, and H3K27ac CUT&Tag data of the K562 cell line generated by the Henikoff laboratory [51] were used to benchmark the performance of narrow-type peak calling software. These are PE data with a read length of 25 bp, and the control IgG-input data of the K562 cell line came from the same publication.

The gold-standard true-positive reference data (H3K4me3, H3K27ac, and CTCF) were defined using K562 ChIP-seq data generated by ENCDOE. Narrow-type peaks of replicates (H3K4me3: ENCSR668LDD, ENCSR000EWA, ENCSR000DWD, and ENCSR000AKU; H3K27ac: ENCFF269RAB, ENCFF546CSD, and ENCFF652VDP; CTCF: ENCFF582SNT, ENCFF660GHM, ENCFF736NYC, and ENCFF769AUF) were downloaded from the ENCODE data portal. The peaks identified in all ChIP-seq replicates were defined as the sensitivity reference dataset. The nonredundant peaks after merging were defined as the specificity reference dataset.

The H3K36me3 CUT&Tag data of mESC TX1072 XO cell line generated by the Schulz laboratory [60] were used to benchmark the performance of broad-type peak calling software. These PE H3K36me3 CUT&Tag data with a read length of 75 bp, and the control IgG-input data were obtained from the same publication. The H3K36me3 CUT&Tag signal density on the gene body of each gene (GENCODE M30) was calculated, and the top 10% of genes with the strongest H3K36me3 enrichment (RPKM ranking) were used as the true-positive reference data to evaluate the sensitivity of peak calling tools. The genes with gene body H3K36me3 enrichment (RPKM > 0.5) were used as specificity reference data.

#### AIAP

AIAP v1.1 was run with the default setting by the pre-build Singularity image for both narrow and broad markers. The control IgG-input data file was not included in the analysis.

#### MACS2

We used four MACS2 combination formats (BAM-IgG, BAM-w/o IgG, BED-IgG, and BED-w/o IgG) to evaluate the performance of MACS2 (v2.2.7.1) in different conditions. The BAM files of histone markers and control IgG-input data were generated by AIAP, as described above. The BED files of histone markers and control IgG-input data were generated using the methylQA “density” function. The *q*-value for peak calling was set as 0.01, and option “--broad” was used when performing peak calling of broad histone marker H3K36me3.

#### ChromHMM

ChromHMM [70] (https://ernstlab.biolchem.ucla.edu/ChromHMM/) is a machine learning software based on the multivariate hidden Markov model that annotates the chromatin states. ChromHMM v1.23 was first run with the BinarizeBAM command to train the model by default using the BAM file generated by AIAP. The LearnModel command was run with the default setting on the binarized files generated in the BinarizeBAM step, learning the chromatin state models to get the annotated regions for the specific markers. The number of states (parameter “numstates”) was set equal to 2 to indicate the binary results produced by ChromHMM. Then, the dense.bed file generated by LearnModel command was processed using awk and cut commands to get the BED3-format BED file as ChromHMM peak calling result. The processing steps were identical for both narrow and broad markers.

#### SEACR

SEACR [71] (https://github.com/FredHutch/SEACR) is a software based on the analysis strategy that uses the global distribution of background signals to calibrate a simple threshold for peak calling. SEACR was designed for CUT&RUN data analysis but was also recommended by its developer for CUT&Tag data analysis. SEACR v1.3 was used to call peaks with the signal and control IgG-input data using bedGraph files generated by methylQA “density” function. Following the developers’ recommendations, the normalization of the control file step was set (parameter “norm”), and the peak calling model was also set to stringent (parameter “stringent”). The peak calling steps were identical for both narrow and broad peak types.

#### CUT&RUNTools 2.0

CUT&RUNTools 2.0 [56] (https://github.com/fl-yu/CUT-RUNTools-2.0) is a flexible pipeline for the analysis and visualization of CUT&Tag and CUT&RUN data at both bulk and single-cell levels. It compacts the different software alternatives for data analysis and provides functions for sequence alignment, QC, dimensionality reduction, cell clustering, data aggregation, and visualization. Here, we used its version published on 09/02/2021 to test the peak calling of bulk CUT&Tag data. The default settings of peak calling were used, including spike_in as FALSE, frag_120 as TRUE, dup_peak_calling as FALSE, the experiment_type as CUT&Tag, and the adapter_type as Truseq. We used the default peak calling parameter (parameter “peak_caller”) to generate all the peak calling results of embedded MACS2-Narrow, MACS2-Broad, and SEACR. The peak calling steps were identical for both narrow and broad peak types.

#### F-Seq

F-Seq v1.85 was used to call peaks using the BED files generated by methylQA “density” function as the input files. The output file format was set to BED file (option “-of”), and the standard deviation cutoff was set to 4 as default. The peak calling steps were identical for both narrow and broad peak types.

#### HMMRATAC

HMMRATAC v1.2.10 was used to call peaks by inputting the trimmed PE BAM files created by AIAP. HMMRATAC was run with “-u” option as 35 and “-l” option as 2 to increase the number of peaks. The “--bedgraph true” option was added to facilitate the downstream analysis and visualization. The peak calling steps were identical for both narrow and broad peak types.

### Differential analysis benchmarking of CUT&Tag data

#### Data used in the study

The data used in this study are listed in Table S1. To benchmark the different strategies of differential analysis and identify the DMPs, we used both narrow-type and broad-type CUT&Tag data. H3K4me3 CUT&Tag data of mESC TX1072 XO cell line on Day 0, Day 2, and Day 4 generated by the Schulz laboratory [60] were used to identify the narrow-type DMPs. H3K36me3 CUT&Tag data of the same cell line at the same sampling time points [60] were used to determine the broad-type DMPs. These data and their input control data were paired-ended with the read length of 75 bp provided in the same experiment.

#### DiffBind

DiffBind [72] (http://bioconductor.org/packages/devel/bioc/html/DiffBind.html) is an R Bioconductor package designed to pinpoint sites with differential enrichment across multiple sample groups. It mainly operates with “peaksets”, which are collections of genomic intervals suggestive of potential protein binding locations for each sample. The package provides various functionalities to manage peaksets, such as overlapping and merging them throughout a dataset, tallying sequencing reads within overlapping intervals of peaksets, and determining statistically significant sites of differential binding, inferred from binding affinity evidence indicated by variations in read densities. The main difference between DiffBind and DESeq2/edgeR is that it allows user to use control IgG-input data for differential analysis. DiffBind v3.4.11 was used to do the tests on R v4.1.2. The general parameters were set equally for the three software, adjusted *P* value or FDR was set equal to 0.01, the log_2_ fold change was set equal to 1, and the CPM filter value was set equal to 1. The method parameter (parameter “method”) was set as DBA_DESEQ2 or DBA_EDGER to get the edgeR and DESeq2 differential analysis results, respectively.

#### edgeR

edgeR [73] (https://bioconductor.org/packages/release/bioc/html/edgeR.html) is one of the most widely used software developed for downstream data analysis for count-based expression data. It assumes that the data can be summarized into a table of counts, with rows corresponding to genes, tags, exons, or transcripts, and columns to samples following the negative binomial distribution. edgeR v3.36.0 and R v4.1.2 were used in the analysis. The FDR cutoff was set equal to 0.01, the log_2_ fold change was set equal to 1, and the CPM filter value was set equal to 1. The normalization step was done by the calNormFactors command with the default setting.

#### DESeq2

DESeq2 [74] (https://bioconductor.org/packages/release/bioc/html/DESeq2.html) is an R Bioconductor package designed for the differential analysis of count data. It utilizes shrinkage estimation for dispersions and fold changes, enhancing the stability and interpretability of the results. It models differential expression using the negative binomial distribution, similar to the approach taken by edgeR. DESeq2 v1.34.0 and R v4.1.2 were used in the analysis. The adjusted *P* value cutoff was set equal to 0.01, the log_2_ fold change was set equal to 1, and the CPM filter value was set equal to 1.

## Code availability

The source code, Docker image, and documentation of this work are freely available at https://github.com/Zhang-lab/Tn5DP.

## CRediT authorship statement

**Siyuan Cheng:** Methodology, Formal analysis, Resources. **Benpeng Miao:** Investigation, Software, Formal analysis, Visualization. **Tiandao Li:** Methodology, Software, Formal analysis, Visualization. **Guoyan Zhao:** Methodology, Investigation, Supervision, Writing – original draft, Writing – review & editing. **Bo Zhang:** Conceptualization, Methodology, Formal analysis, Investigation, Supervision, Project administration, Writing – original draft, Writing – review & editing, Visualization. All authors have read and approved the final manuscript.

## Supplementary Material

qzae054_Supplementary_Data
