# Supplementary material for: Review and Evaluate the Bioinformatics Analysis Strategies of ATAC-seq and CUT&Tag Data
Source: Genomics Proteomics Bioinformatics. 2024 Sep 10;22(3):qzae054. doi: 10.1093/gpbjnl/qzae054 (PMC11464419; doi:10.1093/gpbjnl/qzae054)
Supplement: qzae054_Supplementary_Data [file qzae054_supplementary_data.zip › Supplementary material captions.docx]

**Supplementary material**

**Figure S1 Workflow of data processing and benchmarking**

**Figure S2 Performance comparison of bioinformatics tools used in narrow-type peak CUT&Tag data analysis**

**A.** Left: the number of H3K27ac CUT&Tag peaks in human K562 cells was identified with the tested bioinformatics tools. Right: H3K27ac CUT&Tag peak identification sensitivity and specificity by the tested bioinformatics tools. **B.** Left: the number of H3K4me3 CUT&Tag peaks in mouse ESC cells was identified with the tested bioinformatics tools. Middle: sensitivity and specificity of H3K4me3 CUT&Tag peak identification by the tested bioinformatics tools. Right: peak width distribution of the H3K4me3 peaks identified by the tested bioinformatics tools. **C.** Peak distribution of the CTCF CUT&Tag identified peaks in human K562 overlapped with the ATAC-seq peak and CTCF ChIP-seq identified peaks.

**Table S1 List of datasets used in this study**
