# Supplementary figures and images for: Review and Evaluate the Bioinformatics Analysis Strategies of ATAC-seq and CUT&Tag Data
Source: Genomics Proteomics Bioinformatics. 2024 Sep 10;22(3):qzae054. doi: 10.1093/gpbjnl/qzae054 (PMC11464419; doi:10.1093/gpbjnl/qzae054)

A

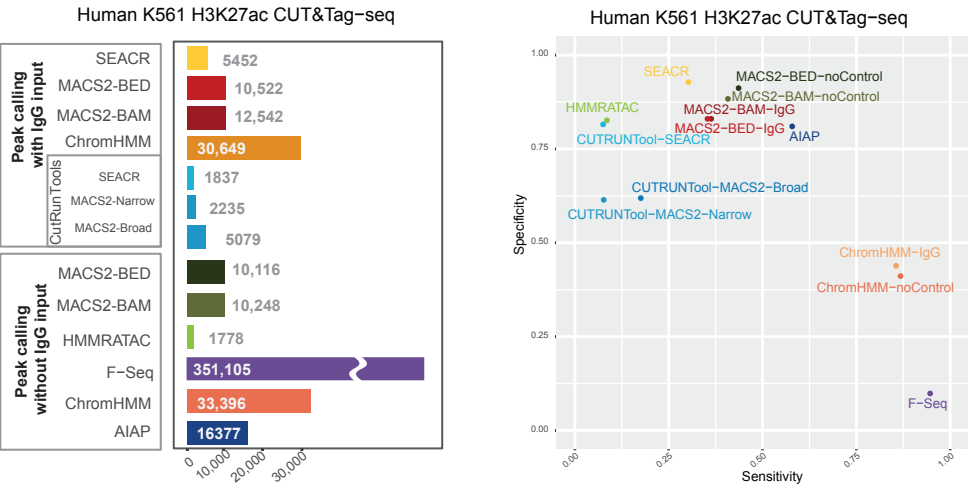

B

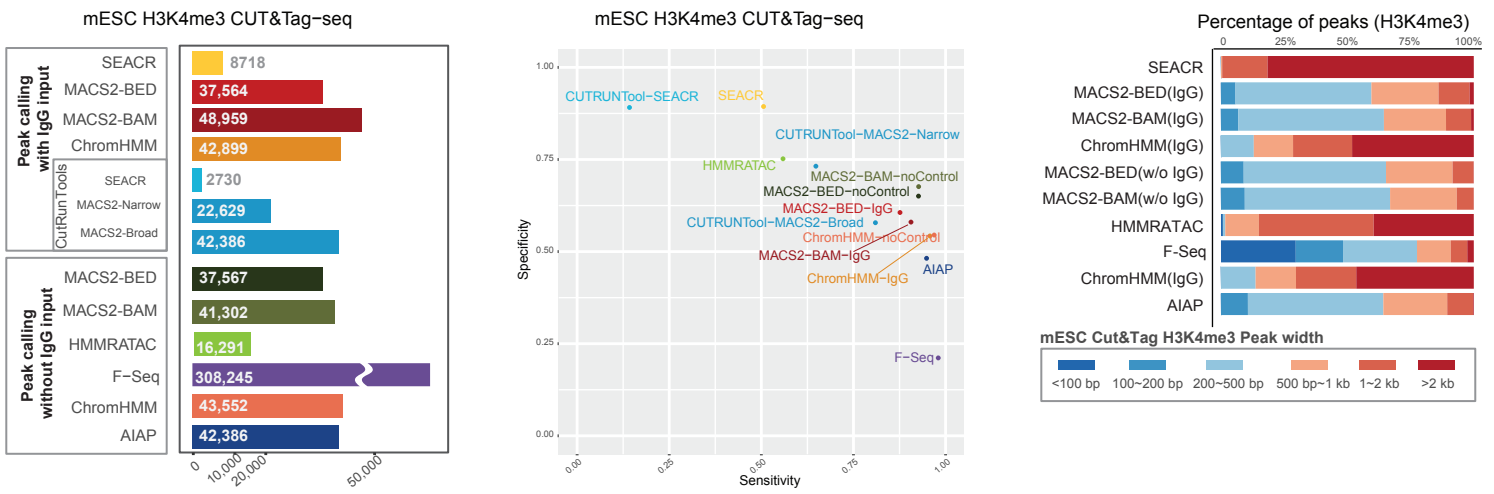

C

### K562 CTCF CUT&Tag-seq peak (by AIAP)

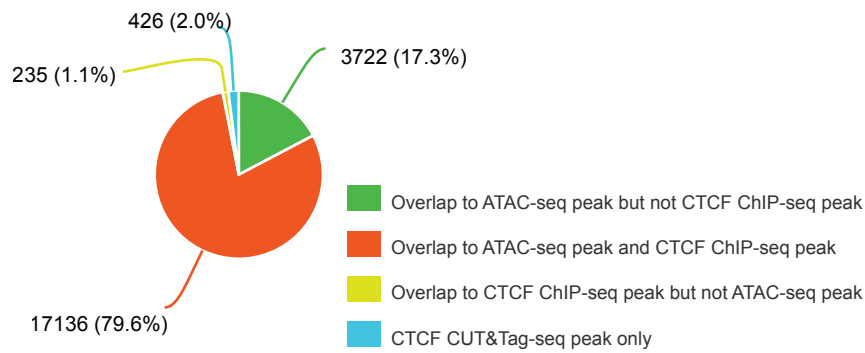

Supplement: qzae054_Supplementary_Data [file qzae054_supplementary_data.zip › Figure S2.pdf]

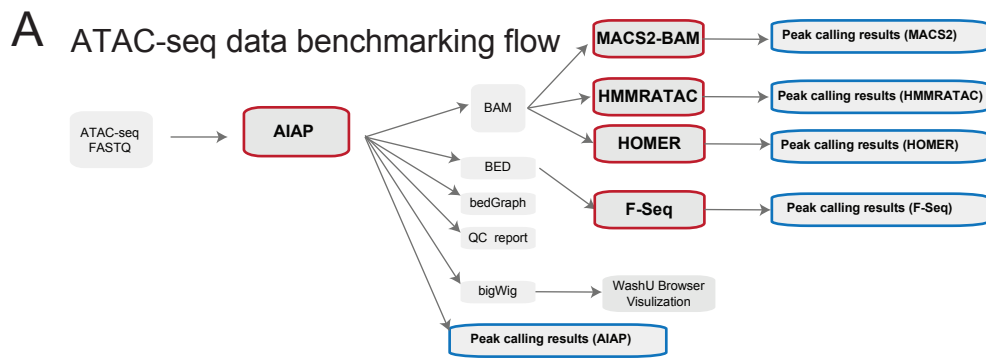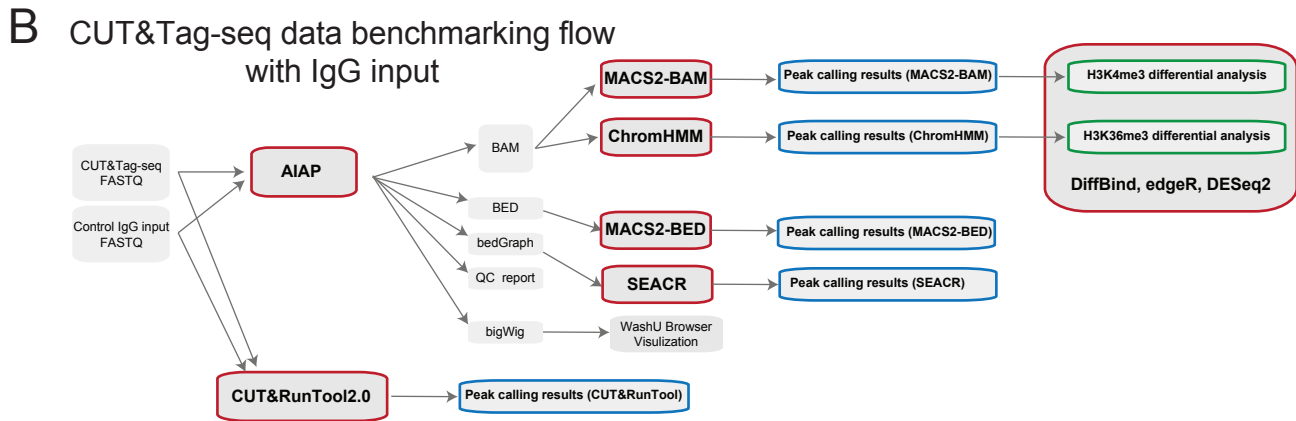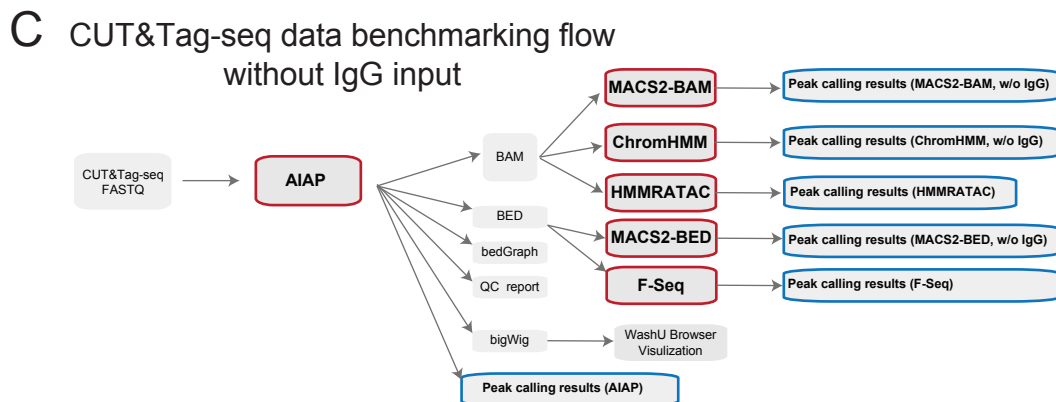

Supplement: qzae054_Supplementary_Data [file qzae054_supplementary_data.zip › Figure S1.pdf]
